# Moth Diversity Increases along a Continent-Wide Gradient of Environmental Productivity in South African Savannahs

**DOI:** 10.3390/insects13090778

**Published:** 2022-08-28

**Authors:** Sylvain Delabye, David Storch, Ondřej Sedláček, Tomáš Albrecht, David Hořák, Vincent Maicher, Anna Tószögyová, Robert Tropek

**Affiliations:** 1 Departments of Ecology and Zoology, Faculty of Science, Charles University, Viničná 7, 12844 Prague, Czech Republic; 2 Institute of Entomology, Biology Centre, Czech Academy of Sciences, Branišovská 31, 37005 České Budějovice, Czech Republic; 3 Department of Zoology, Faculty of Science, University of South Bohemia, Branišovská 1760, 37005 České Budějovice, Czech Republic; 4 Center for Theoretical Study, Charles University, Prague and the Czech Academy of Sciences, Jilská 1, 11000 Prague, Czech Republic; 5 Institute of Vertebrate Biology, The Czech Academy of Sciences, Studenec 122, 67502 Koněšín, Czech Republic; 6 Nicholas School of the Environment, Duke University, 9 Circuit Dr., Durham, NC 27710, USA

**Keywords:** abundance, Afrotropics, Heterocera, insect, diversity patterns, light trapping, lepidoptera, NDVI, primary productivity, savannah ecosystems

## Abstract

**Simple Summary:**

Environmental productivity is considered among the key factors responsible for the uneven distribution of biodiversity on the globe, despite the lack of comprehensive studies for many groups of organisms and regions. We partly filled this gap by this study of moth diversity along the unique continent-wide gradient of environmental productivity across southern African savannahs. We revealed a significantly positive relationship of the moth species richness and environmental productivity, which we did not observe for moth abundance. We hypothesize the effects of water availability, habitat complexity, and plant diversity drive the described relationships.

**Abstract:**

Environmental productivity, i.e., the amount of biomass produced by primary producers, belongs among the key factors for the biodiversity patterns. Although the relationship of diversity to environmental productivity differs among studied taxa, detailed data are largely missing for most groups, including insects. Here, we present a study of moth diversity patterns at local and regional scales along a continent-wide gradient of environmental productivity in southern African savannah ecosystems. We sampled diversity of moths (Lepidoptera: Heterocera) at 120 local plots along a gradient of normalized difference vegetation index (NDVI) from the Namib Desert to woodland savannahs along the Zambezi River. By standardized light trapping, we collected 12,372 specimens belonging to 487 moth species. The relationship between species richness for most analyzed moth groups and environmental productivity was significantly positively linear at the local and regional scales. The absence of a significant relationship of most moth groups’ abundance to environmental productivity did not support the role of the number of individuals in the diversity–productivity relationship for south African moths. We hypothesize the effects of water availability, habitat complexity, and plant diversity drive the observed moth diversity patterns.

## 1. Introduction

Environmental productivity, defined as the rate of biomass production in the ecosystem, ranks among the most studied ecological factors in relation to the global patterns of biodiversity [1,2,3,4,5]. It determines the availability of various resources which have been hypothesized to drive the intensity of interspecific competition [6] and to limit the number of coexisting species [7,8]. Additionally, the species–energy theory assumes that the habitat’s energy supply sets an upper limit to the number of individuals in a community [7], and the more individuals hypothesis (MIH) has been suggested to explain the positive linear relationship of species richness with environmental productivity [7,9]. It expects that low productivity cannot support a high number of species, since these communities would have such small populations that their extinction rates would be higher than origination rates [10]. Although several studies found that species richness has often been positively related to available energy, diversity patterns do not seem to be mediated by the number of individuals [3,11,12].

However, the relationship of animal diversity with environmental productivity (or its surrogates; see [13]) has been unevenly studied, and therefore, its general patterns remain unclear and inconsistent, especially for some groups (e.g., [1,2,5]). The existing studies and reviews revealed either linearly increasing diversity with productivity or a hump-shaped relationship with the highest diversity in the intermediately productive environments (e.g., [1,2,5]), although decreasing diversity with environmental productivity and non-significant relationships were also found (e.g., [1,2]). Some authors also suggested that the relationship varies across geographic scales (e.g., [1,2,14]). While the diversity can show the hump-shaped patterns at local scales, it increases mostly linearly with environmental productivity at regional or larger scales [14]. Nevertheless, such rules are by no means universal, as Cusens et al. [15] found no support for the scale dependency of the patterns in their meta-analysis, and the significant, positive linear relationship prevailed across the scales.

For diversity and abundance spatial patterns of insects, one of the most abundant and speciose groups in terrestrial ecosystems, environmental productivity has repeatedly been suggested as the key driver [16,17], despite the general lack of available data [9,11]. Moreover, most of the few available studies included the above-mentioned bias of the confounding effects of environmental productivity with latitude or elevation. When environmental productivity was studied independently of elevation and latitude, a significant positive linear relationship was found for butterflies [18,19], ants [20], and damselflies [21]; and marginally positive or non-significant relationships were found for butterflies [22,23] and aquatic insects [24], at both local and regional scales. In the Afrotropics, only the diversity patterns of sphingid moths were studied at larger scales, evaluating environmental productivity as one of the main responsible variables, with a strong positive correlation with species richness [25]. Nevertheless, this study relied on the diversity data from modelled species distribution, and any relationship with environmental variables could thus be artificial, as the variables had been used for the modelling as well. This highlights how poorly the drivers of insect diversity patterns were studied in the Afrotropics, particularly in the Afrotropical savannahs [26].

**Figure 1:**
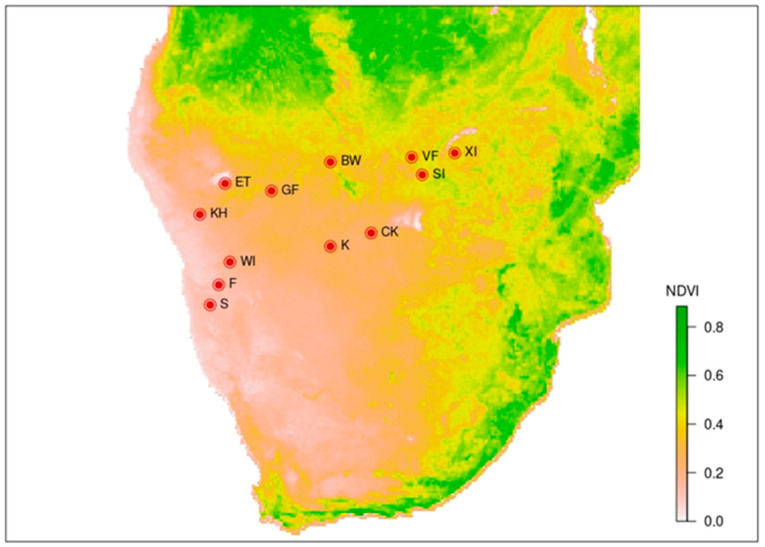
Regions in southern Africa sampled for moth communities along the gradient of environmental productivity. Mean NDVI in the beginning of vegetation season (October to December) is visualized. Region codes are listed in Table 1.

In this study, we focus on patterns of diversity and abundance of adult moths along a latitude-unrelated, continent-wide gradient of environmental productivity in southern African savannahs, at the local and regional scales. The studied gradient of environmental productivity (Figure 1) is unique in its high independency on other climate variables, especially environmental temperature, to whose gradient it is more or less perpendicular [27]. Moths are a diverse group of commonly used biodiversity indicators, with an important role in ecosystem food webs, including herbivory, prey for many predators and pollination. As mostly primary consumers, their communities can be expected to be closely related to environmental productivity. We specifically asked the following questions: (1) Do relationships between species richness and environmental productivity differ at local (alpha diversity) and regional (gamma diversity) scales? (2) How is abundance related to environmental productivity at both scales? We predicted a positive linear relationship of species richness and abundance of moths to environmental productivity at both scales. To better understand the revealed patterns, we also performed partial analyses for abundant moth subgroups.

## 2. Materials and Methods

### 2.1. Data Sampling

The field sampling was conducted along a continent-wide gradient of environmental productivity in southern Africa, from the deserts in western Namibia, through semideserts and open savannahs in Namibia and Botswana, to productive woodland savannahs in northwestern Zimbabwe [28]. Along this productivity gradient, we sampled moth communities in 12 regions in open and semi-open natural habitats (Figure 1; Table 1).

In each sampling region, 10 local plots were selected at least 1 km apart from each other, forming a 10 km transect, or two perpendicular transects in some regions. Nocturnal moths were collected using portable light traps (with 48 LED lights arranged into two strips, prevailing UV light spectrum—400 nm, 400 lm; run by a 12 V battery). The sampling was carried out during the beginning of the vegetation season, i.e., November and December (see Table S1 for particular sampling dates). The nights with forecasted temperature drop or strong wind were avoided. To decrease the effect of weather on the moth capture efficiency, sampling of individual plots in each region was split in two or three nights, whenever possible. All captured moths were euthanized by ammonium carbonate placed in a small mesh bag in each trap. A light trap was exposed for a night (from dusk till dawn) at individual local sampling plots. Moth specimens were sorted out in the field, dried by silica gel, and stored in paper envelopes. All individuals of the target moth groups (Noctuoidea: Erebidae, Eutellidae, Noctuidae, Notodontidae; Bombycoidea *s.l.*: Eupterotidae, Lasiocampidae, Saturniidae, Sphingidae—hereinafter referred to as Bombycoidea; Zygaenoidea: Limacodidae) were later mounted; identified by species or morphospecies through a combination of morphological characters and genitalia dissections; and counted. Specimens of Geometroidea were counted but not identified (especially because numerous specimens of tiny geometrid species would require intensive genitalia dissections which was not within our capabilities); therefore, this superfamily was used only for analyses of abundances but not for analyses of species richness. The voucher material is stored in the Biology Centre, Czech Academy of Sciences, České Budějovice, Czechia.

Environmental productivity was characterized by the normalized difference vegetation index (NDVI) for quantifying remotely sensed vegetation greenness. NDVI is a widely accepted proxy for environmental productivity, commonly applied at different spatial scales in order to predict species richness [13]. We used the NDVI values produced by an extended, 8 km Advanced Very High Resolution Radiometer (AVHRR; [29]). We used the average of monthly maximum NDVI from the beginning of the vegetation season in the studied region (i.e., from October to December) from years 1982–2004 [29]. Each local sampling plot was characterized by three measures of environmental productivity (maximum, minimum, and mean NDVI) of its 8 km grid cell. Moreover, to partly describe the habitat complexity, individual vegetation layer coverages were visually estimated at each local plot during the setting of the light traps. For the regional-scale analyses, the values of each characteristic were averaged for the 10 local plots of each region (Table 1). We tested collinearity among all described characteristics. As virtually all characteristics were intercorrelated (Pearson ρ ≥ |0.5|; Table S2), we selected mean NDVI as the only proxy for environmental productivity in our analyses.

### 2.2. Data Analyses

We analyzed the relationship of moth diversity with environmental productivity in R 4.0.3 [30]. All analyses were first run with the complete datasets (i.e., all moths for abundances, and all moths except Geometroidea for species richness), followed by separate analyses of particular moth groups to reveal potentially different patterns among them. Based on the numbers of sampled species and specimens (Table 2), superfamilies Bombycoidea and Noctuoidea were analyzed separately also. As families Erebidae and Noctuidae (both belonging to the Noctuoidea superfamily) were substantially abundant in our material, and they are common focal groups for diversity studies, we ran separate analyses for them as well.

We tested the relationships of *alpha diversity* (mean number of species sampled at individual local plots in each sampling region), *gamma diversity* (number of species sampled at all local plots in each region), and *abundance* (number of all specimens at all local plots in each region) with environmental productivity (mean NDVI) by linear models (after visual checking for the normal distribution in our data). As unimodal models were found as one of the main patterns on the local scale in some other studies (see above), we also tested the relationships by unimodal models. To allow better comparison with other studies of moth communities, we also calculated Fisher’s-α diversity indices. Nevertheless, as we did not have any hypotheses for the relationship between environmental productivity and the shape of species-abundance distribution (represented by Fisher’s-α index; [31]), we did not include them in our analyses.

## 3. Results

In total, 12,372 individuals of the focal moth groups were captured. Among these, 9048 individuals were identified of 487 species or morphospecies (Table 2 and Table S3), and 3324 specimens of Geometroidea were counted without further identification. Fisher’s-α indices of the sampled moth communities ranged between 3.89 and 140.71 at the regional scale, and 1.19 and 23.62 at the local scale (Table S4).

**Figure 2:**
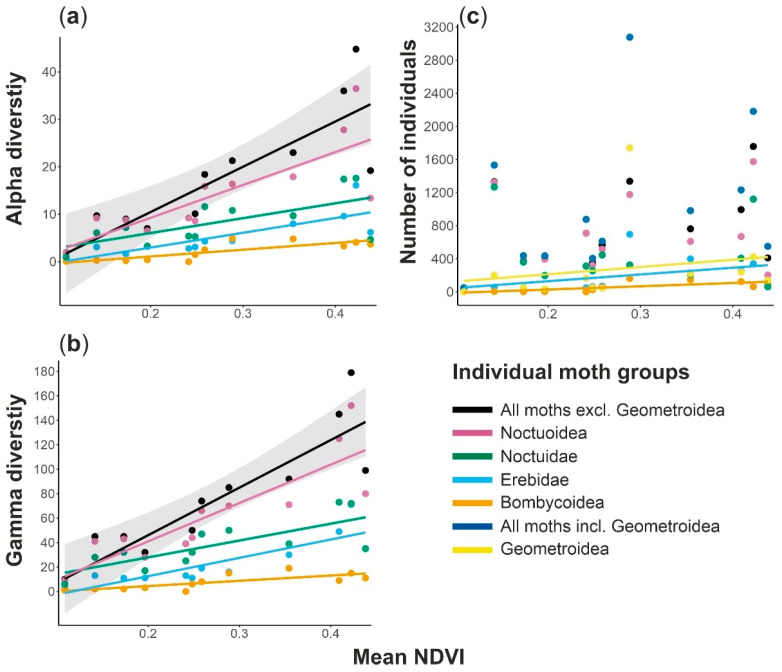
Effects of environmental productivity (mean NDVI) on (**a**) alpha diversity (i.e., mean local species richness), (**b**) gamma diversity (i.e., regional species richness), and (**c**) numbers of individuals (i.e., abundance) in individual moth groups in southern Africa. Only the significant relationships are visualized; see Table 3 for all models results. Shaded areas indicate 95% confidence intervals for the main models with all target moth groups pooled.

All studied groups showed significant positive linear relationships of alpha and gamma diversities with mean NDVI, whereas the unimodal relationships were non-significant for all models (Table 3, Figure 2a,b and Figure S1). For alpha diversity, the coefficients of determination R^2^ were greater than 60% for linear models of all moth groups but Noctuidae. All moths, excluding Geometroidea, showed higher R^2^ for both alpha and gamma diversity (68% and 76%, respectively) than all partial models for individual moth groups.

Abundances of all moths, and most of the analyzed moth groups, showed non-significant relationships with mean NDVI, except Bombycoidea, Geometroidea, and Noctuidae, which showed significant positive linear relationships (Table 3, Figure 2c). Nevertheless, these correlations were relatively weak for Geometroidea and Noctuidae (R^2^ = 37% and 28%, respectively). The abundance of Bombycoidea correlated with mean NDVI relatively better (R^2^ = 57%). No significant unimodal relationships of abundance and environmental productivity were detected.

## 4. Discussion

The hypothesized linear increase in species richness along the environmental productivity gradient was confirmed for the local (alpha diversity) and regional (gamma diversity) scales for all analyzed moth groups. Nevertheless, a significantly positive relationship for moth abundance was found for three studied moth groups only, i.e., Geometroidea, Bombycoidea, and Erebidae, all with relatively low amounts of explained variability (Table 3). The non-significant patterns were shown for all moths, and for Noctuoidea and Noctuidae.

Our study confirmed environmental productivity as the driver of moth diversity in southern African savannahs. This finding is concordant with the results of large-scaled studies of butterfly diversity in the North American Great Basin [18,19], and of Afrotropical hawkmoths [25]. Environmental productivity also played a key role in global diversity patterns of ants [20], in the diversity of damselflies in the Amazon [21], and in the diversity of freshwater invertebrates (including insects) in ponds across 10 watersheds [14]. However, it did not significantly affect butterfly diversity across Canada, where habitat heterogeneity crucially explained the diversity and community composition of butterflies [22]. Nevertheless, the positive relationship of species richness with environmental productivity seems to prevail in insects, as supported by our results, although more data for various groups and from more areas would be needed to confirm the general pattern and to analyze its causes.

Although our results were consistent for both analyzed scales, the small-scaled relationship of local diversity and environmental productivity vary among insect taxa and regions in the available studies. Consistently with our results, environmental productivity was crucial for butterfly diversity along an elevational gradient of Mount Hernon in Israel [32]. However, on the elevational gradient of Mount Kilimanjaro, springtails and ground-dwelling beetles were the only insect groups with species richness positively related to environmental productivity [33]. Diversity of various hymenopterans showed a negative relationship, whereas species richness of moths, hoverflies, orthopterans, hemipterans, parasitoid wasps, and dung beetles had no significant relationship with environmental productivity [33]. Moreover, Chase and Leibold [14] suggested that the relationship of diversity with environmental productivity at a local scale should be expected to be hump-shaped, based on pond invertebrates, including insects. Nevertheless, such a pattern was not confirmed by any other study on insects, including our data on Afrotropical moths.

Our study did not confirm the more individual hypothesis (MIH). Due to numerous methodological constraints (e.g., [34,35]), insect abundance in communities is rarely analyzed in multi-species studies. The existing studies showed no relationship of butterfly abundance with environmental productivity across Northern America [11], and a positive relationship of ant abundance at a global scale [20]. Nevertheless, quantification of insect abundance requires more effort, especially due to the strong interannual fluctuations [35], which has not been done in any existing study, including ours. Therefore, despite the various problems of MIH [10], its validity for insects cannot be elaborately analyzed yet.

Generally, temperature was often hypothesized or even evidenced as being among the important factors influencing the diversity of ectotherms, including insects, along the environmental productivity gradients [3,5,7,9]. Nevertheless, it is often confounded with other correlates of environmental productivity because most diversity patterns have been studied along latitudinal or elevational gradients [13]. Although we have not tested this relationship, the mean temperature is known not be correlated with environmental productivity in southern Africa (and we intentionally selected our localities to be independent of environmental temperature; Figure 1), showing more complex gradients in the region [27]. Therefore, we cannot confirm that temperature can have positive effects on the diversity of south African moths. Similarly, a negative diversity–temperature relationship was documented for plants in south African savannahs [36,37].

On the other hand, precipitation, as the second of the commonly hypothesized important drivers, is positively correlated with environmental productivity in southern Africa [27,38]. Therefore, we speculate that water availability is the key component of productivity responsible for the observed southern African moth diversity patterns [39]. Insect diversity is well-known to increase in environments with complex and heterogeneous habitats [16,22,40,41,42]. We showed an increase in vegetation cover in all its layers with environmental productivity along the sampled gradient (Table S2), which supports this hypothesis.

Finally, the diversity of insects was repeatedly proven to correlate with the diversity of plants [17,42,43]. Although the relationship of plant diversity with environmental productivity may vary regionally (e.g., [44,45]), environmental productivity has recently been proven as the key driver of global plant diversity [5]. In south African savannahs, the positive diversity–productivity relationship was shown for woody plants [36], the key plant group for diversity of Afrotropical moths [42]. Altogether, we hypothesize that the observed increase in moth diversity along the environmental productivity gradient can be related to changes in water availability causing increases in diversity of plants and complexity of habitats.

## Figures and Tables

**Table 1:** Summary and characteristics of the regions (with their codes used in Figure 1) sampled for moth diversity along the environmental productivity gradient in southern Africa. The NDVI values and vegetation layer coverages were averaged from the 10 plots for each region.

Region (Code)	Elevation (m a.s.l.)	Latitude/ Longitude	Habitat Type	Max./ Mean/ Min. NDVI	Vegetation Cover (%)				
					All	30 cm	2 m	5 m	>5 m
**Soussusvlei (S)**	760	S 24.543° E 15.789°	Namib Desert with very scarce vegetation	0.1119 0.1086 0.1038	14.6	9.9	4.1	0.5	0.0
**Namibgrens (F)**	1790	S 23.643° E 16.279°	Namib Escarpment Woodland: dry savannahs and shrubby areas with scattered trees	0.1448 0.1417 0.1383	16.8	6.8	5.3	4.7	0.0
**Khorixas (KH)**	1040	S 20.440° E 15.215°	Angolian Mopane Woodland: mosaic of *Acacia* and mopane woodlands	0.1864 0.1732 0.1593	45.7	2.8	22.3	17.7	2.9
**Windhoek (WI)**	1800	S 22.608° E 16.773°	Namib Escarpment Woodland: dry savannahs and shrubby areas with scattered trees	0.2276 0.1964 0.1719	46.9	26.0	16.2	4.7	0.0
**Etosha (ET)**	1120	S 19.051° E 16.541°	Angolian Mopane Woodland: mosaics of *Acacia* and mopane woodlands	0.2837 0.2412 0.2018	93.7	39.4	32.7	21.6	0.0
**Thakadu (K)**	1120	S 21.867° E 21.697°	Kalahari Xeric Savannah: dry open savannahs, with scattered trees	0.2979 0.2481 0.2047	106.0	35.5	49.5	20.5	0.5
**Central Kalahari (CK)**	980	S 21.288° E 23.716°	Kalahari *Acacia* Woodland: mosaics of *Vachellia*, *Baikeia* and mopane woodlands, and small-leaved savannahs	0.3487 0.2586 0.1912	87.7	26.0	33.5	23.5	4.7
**Grootfontein (GF)**	1220	S 19.346° E 18.812°	Kalahari Acacia Woodland: mosaics of *Vachellia*, *Baikeia* and mopane woodlands, and small-leaved savannahs	0.3554 0.2884 0.2264	133.2	55.0	45.0	27.5	5.7
**Bwabwata (BW)**	1030	S 18.092° E 21.686°	Zambezian *Baikiaea* Woodlands: mosaic of mopane and *Baikeia* woodlands, and secondary grasslands	0.4459 0.3542 0.2689	114.6	39.5	40.3	12.5	22.3
**Hwange (SI)**	1020	S 18.699° E 26.192°	Zambezian and Mopane Woodlands: mosaic of miombo and mopane woodlands, and shrubby savannahs	0.5468 0.4090 0.2734	106.1	42.3	35.5	18.0	10.3
**Victoria Falls (VF)**	920	S 17.872° E 25.721°	Zambezian and Mopane Woodlands: mosaic of mopane and *Baikeia* woodlands, and secondary grasslands	0.5431 0.4222 0.3066	123.8	49.0	41.5	25.2	8.1
**Chizarira (XI)**	1010	S 17.701° E 27.855°	Zambezian and Mopane Woodlands: mosaic of mopane and *Baikeia* woodlands, and secondary grasslands	0.5692 0.4379 0.3145	124.2	45.2	27.2	32.0	15.1

**Table 2:** Diversity of the focal moth groups at individual regions: gamma diversity (γ: regional species richness), alpha diversity (α: mean local species richness), abundance (Ab.: regional number of specimens).

**Region**	**All Moths Exc. Geometroidea**			**Bombycoidea**			**Noctuoidea**			**Erebidae**			**Noctuidae**			**All Moths Incl. Geometroidea**			**Geometroidea**		
	**γ**	**α**	**Ab.**	**γ**	**α**	**Ab.**	**γ**	**α**	**Ab.**	**γ**	**α**	**Ab.**	**γ**	**α**	**Ab.**	**Ab.**			**Ab.**		
Soussusvlei	10	1.9	47	1	0.1	1	9	1.8	46	3	0.8	23	6	1.0	23	50			3		
Namibgrens	45	9.7	1331	2	0.3	5	41	9.2	1324	13	3.1	57	28	6.1	1267	1532			201		
Khorixas	45	9.0	382	2	0.2	2	43	8.8	380	11	1.6	20	32	7.2	360	438			56		
Windhoek	32	7.0	399	3	0.4	4	28	6.5	394	11	3.2	195	17	3.3	199	436			37		
Etosha	39	9.2	710	0	0.0	0	39	9.2	710	13	2.8	50	25	5.4	312	876			166		
Thakadu	50	10.1	350	6	1.5	27	44	8.6	323	11	3.1	64	32	5.3	254	404			54		
Central Kalahari	74	18.4	569	8	2.5	54	66	15.9	515	19	4.3	69	47	11.6	446	612			43		
Grootfontein	85	21.3	1337	15	4.9	161	70	16.4	1176	16	4.4	696	50	10.8	325	3078			1741		
Bwabwata	92	23.0	762	19	4.8	149	71	17.9	610	30	8.0	399	39	9.7	209	982			220		
Hwange	145	36.0	994	9	3.3	124	125	27.8	669	49	9.6	249	73	17.4	406	1232			238		
Victoria Falls	179	44.8	1757	15	4.1	63	152	36.5	1574	71	16.1	340	72	17.6	1121	2182			425		
Chizarira	99	19.2	410	11	3.7	98	80	13.4	203	35	6.2	102	35	4.7	62	550			140		
**Region**	**Eutellidae**			**Lasiocampidae**			**Limacodidae**			**Notodontidae**			**Saturniidae**			**Sphingidae**			**Eupterotidae**		
	**γ**	**α**	**Ab.**	**γ**	**α**	**Ab.**	**γ**	**α**	**Ab.**	**γ**	**α**	**Ab.**	**γ**	**α**	**Ab.**	**γ**	**α**	**Ab.**	**γ**	**α**	**Ab.**
Soussusvlei	0	0	0	1	0.1	1	0	0	0	0	0	0	0	0	0	0	0	0	0	0	0
Namibgrens	0	0	0	2	0.3	5	2	0.2	2	0	0	0	0	0	0	0	0	0	0	0	0
Khorixas	0	0	0	0	0	0	0	0	0	0	0	0	1	0.1	1	1	0.1	1	0	0	0
Windhoek	0	0	0	2	0.2	2	1	0.1	1	0	0	0	0	0	0	0	0	0	1	0.2	2
Etosha	1	1.0	348	0	0	0	0	0	0	0	0	0	0	0	0	0	0	0	0	0	0
Thakadu	0	0	0	2	0.9	18	0	0	0	1	0.2	5	1	0.1	1	2	0.3	4	1	0.2	4
Central Kalahari	0	0	0	4	1.5	37	0	0	0	0	0	0	1	0.1	1	3	0.9	16	0	0	0
Grootfontein	1	0.1	1	6	2.1	94	0	0	0	3	1.1	154	1	0.2	2	8	2.6	65	0	0	0
Bwabwata	1	0.1	1	7	1.5	47	2	0.3	3	1	0.1	1	4	1.0	26	7	1.9	67	1	0.4	9
Hwange	0	0	0	2	0.3	3	11	4.9	201	3	0.8	14	1	0.2	2	4	1.3	44	2	1.5	75
Victoria Falls	0	0	0	5	1.1	11	12	4.2	120	9	2.8	113	2	0.2	2	5	1.9	35	3	0.9	15
Chizarira	0	0	0	5	1.6	60	8	2.1	109	10	2.5	39	1	0.4	5	2	0.4	4	3	1.3	29

**Table 3:** Results of linear and unimodal models for relationship of moth diversity indexes (alpha diversity, gamma diversity, and abundance) to mean NDVI for each focal moth group. Coefficients of determination (R^2^) are indicated, with the model *p*-values (^n.s.^ *p* ≥ 0.05, * *p* < 0.05; ** *p* < 0.01, *** *p* < 0.001).

	Alpha Diversity		Gamma Diversity		Abundance	
	Linear	Unimodal	Linear	Unimodal	Linear	Unimodal
All moths exc. Geometroidea	0.68 ***	0.76 ^n.s.^	0.75 ***	0.77 ^n.s.^	0.19 ^n.s.^	0.26 ^n.s.^
All moths incl. Geometroidea	-	-	-	-	0.20 ^n.s.^	0.29 ^n.s.^
Geometroidea	-	-	-	-	0.28 *	0.34 ^n.s.^
Bombycoidea	0.63 **	0.66 ^n.s.^	0.51 **	0.47 ^n.s.^	0.57 **	0.56 ^n.s.^
Noctuoidea	0.60 **	0.69 ^n.s.^	0.71 ***	0.73 ^n.s.^	0.03 ^n.s.^	0.15 ^n.s.^
Erebidae	0.63 **	0.77 ^n.s.^	0.82 ***	0.80 ^n.s.^	0.37 *	0.40 ^n.s.^
Noctuidae	0.39 *	0.45 ^n.s.^	0.51 **	0.60 ^n.s.^	−0.08 ^n.s.^	−0.05 ^n.s.^

## Data Availability

The data presented in this study are available on request from the corresponding authors.

## Supplementary Materials

The following supporting information can be downloaded at: https://www.mdpi.com/article/10.3390/insects13090778/s1. Table S1: Sampling dates for individual regions. Table S2: Pearson correlation coefficients among the three measures of environmental productivity, and four characteristics of vegetation cover. Table S3: Overview of the total numbers of identified species and specimens the focal moth groups. Table S4: Fisher-α diversity. Figure S1: Relationships of alpha and gamma diversities of moths with environmental productivity on log-scales.
