# Supplementary material for: Moth Diversity Increases along a Continent-Wide Gradient of Environmental Productivity in South African Savannahs
Source: Insects. 2022 Aug 28;13(9):778. doi: 10.3390/insects13090778 (PMC9500993; doi:10.3390/insects13090778)

Moth diversity increases along a continent-wide gradient of environmental productivity in south African savannahs

SUPPLEMENTARY INFORMATION

Sylvain Delabye[1,2,3,], David Storch[1,4], Ondřej Sedláček[1], Tomáš Albrecht[1,5], David Hořák[1], Vincent Maicher[1,2,6], Anna Tószögyová[1,4], Robert Tropek[1,2,*]*
1 Departments of Ecology and Zoology, Faculty of Science, Charles University, Viničná 7, 12844 Prague, Czechia
2 Institute of Entomology, Biology Centre, Czech Academy of Sciences, Branišovská 31, 37005 České Budějovice, Czechia
3 Department of Zoology, Faculty of Science, University of South Bohemia, Branišovská 1760, 37005 České Budějovice, Czechia
4 Center for Theoretical Study, Charles University, Prague and the Czech Academy of Sciences, Jilská 1, 11000 Praha, Czechia
5 Institute of Vertebrate Biology, the Czech Academy of Sciences, Studenec 122, 67502 Koněšín, Czechia
6 Nicholas School of the Environment, Duke University, 9 Circuit Dr., Durham, NC 27710, USA
**Correspondence: sylvain.delabye@gmail.com (Sylvain Delabye); robert.tropek@gmail.com (Robert Tropek)*

Table S1. Dates of moth sampling in individual localities along an environmental productivity gradient in southern Africa.

Region	Sampling dates
**Soussusvlei**	5th–8th Nov 2016
**Namibgrens**	3th–5th Nov 2016
**Khorixas**	8th–10th Nov 2016
**Windhoek**	9th–12th Nov 2016
**Etosha**	9th–12th Nov 2016
**Thakadu**	13th–16th Nov 2016
**Central Kalahari**	17th–20th Nov 2016
**Grootfontein**	14th–17th Nov 2016
**Bwabwata**	17th–22nd Nov 2016
**Hwange**	9th–11th Dec 2017
**Victoria Falls**	13th–14th Dec 2017
**Chizarira**	5th–6th Dec 2016

Table S2. Pearson correlation coefficients among the three measures of environmental productivity, and four measures of vegetation cover the sampling regions. The variables were considered collinear if Pearson ρ ≤ |0.5| (**in bold**).

	Maximum NDVI	Mean NDVI	Minimum NDVI	Mean vegetation Cover	< 30 cm vegetation layer cover (%)	< 2 m vegetation layer cover (%)	< 5 m vegetation layer cover (%)
Mean NDVI	1.00						
Minimum NDVI	0.98	0.99					
Mean vegetation Cover	0.85	0.86	0.88				
< 30 cm vegetation layer cover (%)	0.80	0.81	0.83	0.93			
< 2 m vegetation layer cover (%)	0.64	0.65	0.67	0.90	0.78		
< 5 m vegetation layer cover (%)	0.70	0.70	0.72	0.84	0.69	0.73	
>5 m vegetation layer cover (%)	0.75	0.76	0.76	0.60	0.50	0.40	0.35

Table S3. Overview of the total numbers of identified species and specimens the focal moth groups sampled along the environmental productivity gradient in southern Africa.

	No. species	No. specimens
**All moth families exc. Geometroidea**	487	9048
**All moth families incl. Geometroidea**	-	12372
**Geometroidea**	-	3324
**Bombycoidea**	44	688
**Noctuoidea**	424	7924
**Erebidae**	153	2264
**Eupterotidae**	5	134
**Eutellidae**	1	350
**Lasiocampidae**	20	278
**Limacodidae**	19	436
**Noctuidae**	251	4984
**Notodontidae**	19	326
**Saturniidae**	8	40
**Sphingidae**	11	236

Table S4. Fisher-α diversity indices of moth communities in individual sampling regions.

**Region**	**Regional Fisher-α**	**Local Fisher-α**
**Soussusvlei**	3.89	1.19
**Namibgrens**	8.99	2.46
**Khorixas**	13.25	6.01
**Windhoek**	8.19	3.08
**Etosha**	8.87	3.30
**Thakadu**	15.96	6.11
**Central Kalahari**	22.69	11.76
**Grootfontein**	20.20	7.44
**Bwabwata**	27.36	13.17
**Hwange**	46.72	23.62
**Victoria Falls**	49.86	20.10
**Chizarira**	140.71	16.13

Figure S1. Effects of environmental productivity (mean NDVI) on log-transformed alpha (i.e. mean local species richness) and gamma (i.e. regional species richness) diversities of individual moth groups in southern Africa.

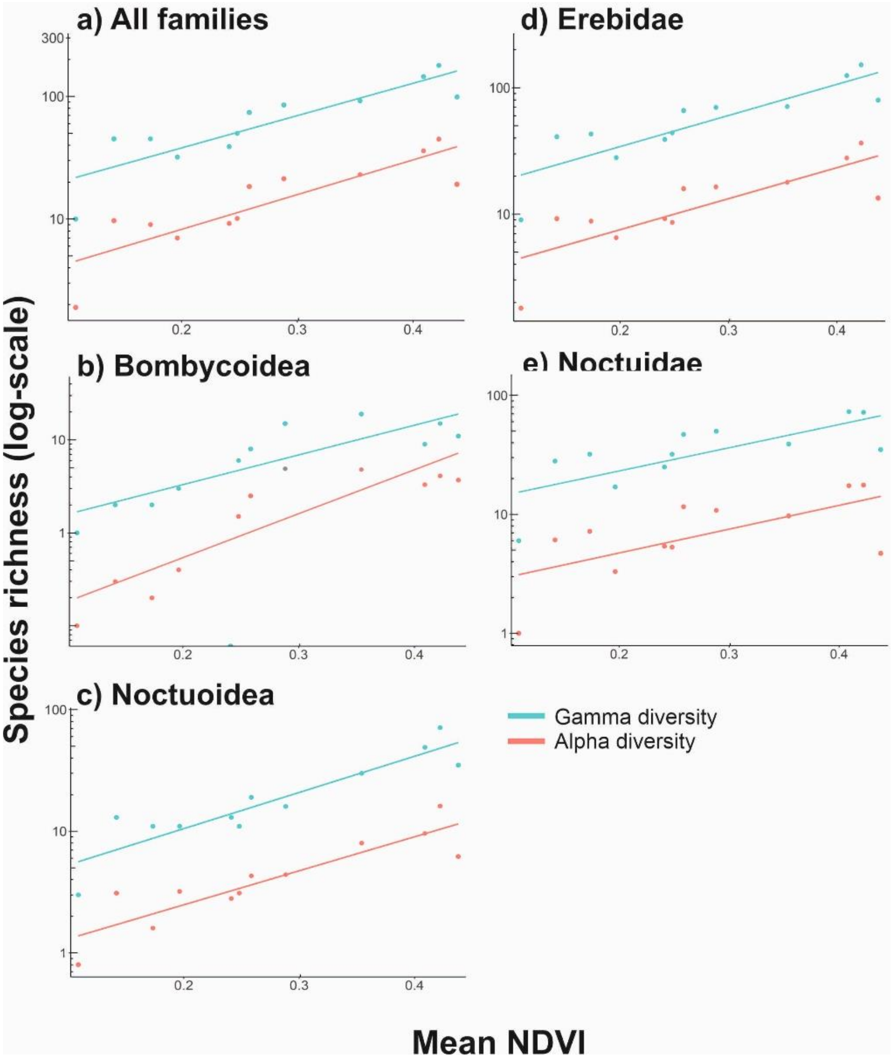

Supplement: Supplementary file 1 [file insects-13-00778-s001.zip › insects-1794318-supplementary.pdf]
